# CXCL12/CXCR4 signaling contributes to neuropathic pain via central sensitization mechanisms in a rat spinal nerve ligation model

**DOI:** 10.1111/cns.13128

**Published:** 2019-04-07

**Authors:** Zhi‐Yuan Liu, Zhi‐Wen Song, Shi‐Wu Guo, Jun‐Sheng He, Shen‐Yu Wang, Jian‐Guo Zhu, Hui‐Lin Yang, Jin‐Bo Liu

**Affiliations:** ^1^ Department of Spinal Surgery The Third Affiliated Hospital of Soochow University Changzhou China; ^2^ Department of Orthopedics The Affiliated Wujin Hospital of Jiangsu University Changzhou China; ^3^ Department of Orthopedics The First Affiliated Hospital of Soochow University Suzhou China

**Keywords:** central sensitization mechanisms, CXCL12, CXCR4, neuropathic pain, spinal nerve ligation

## Abstract

**Background:**

Previous studies have demonstrated that the CXCL12/CXCR4 signaling axis is involved in the regulation of neuropathic pain (NP). Here, we performed experiments to test whether the CXCL12/CXCR4 signaling pathway contributes to the pathogenesis of neuropathic pain after spinal nerve ligation (SNL) via central sensitization mechanisms.

**Methods:**

Neuropathic pain was induced and assessed in a SNL rat model. The expression and distribution of CXCL12 or CXCR4 were examined by immunofluorescence staining and western blot. The effects of CXCL12 rat peptide, CXCL12 neutralizing antibody, CXCR4 antagonist, and astrocyte metabolic inhibitor on pain hypersensitivity were explored by behavioral tests in naive or SNL rats. We measured the expression level of c‐Fos and CGRP to evaluate the sensitization of neurons by RT‐PCR. The activation of astrocyte and microglia was analyzed by measuring the level of GFAP and iba‐1. The mRNA levels of the pro‐inflammatory cytokines such as TNF‐α, IL‐1β, and IL‐6 and Connexin 30, Connexin 43, EAAT 1, EAAT 2 were also detected by RT‐PCR.

**Results:**

First, we found that the expression of CXCL12 and CXCR4 was upregulated after SNL. CXCL12 was mainly expressed in the neurons while CXCR4 was expressed both in astrocytes and neurons in the spinal dorsal horn after SNL. Moreover, intrathecal administration of rat peptide, CXCL12, induced hypersensitivity in naive rats, which was partly reversed by fluorocitrate. In addition, the CXCL12 rat peptide increased mRNA levels of c‐Fos, GFAP, and iba‐1. A single intrathecal injection of CXCL12 neutralizing antibody transiently reversed neuropathic pain in the SNL rat model. Consecutive use of CXCL12 neutralizing antibody led to significant delay in the induction of neuropathic pain, and reduced the expression of GFAP and iba‐1 in the spinal dorsal horn. Finally, repeated intrathecal administration of the CXCR4 antagonist, AMD3100, significantly suppressed the initiation and duration of neuropathic pain. The mRNA levels of c‐Fos, CGRP, GFAP, iba‐1, and pro‐inflammatory cytokines, also including Connexin 30 and Connexin 43 were decreased after injection of AMD3100, while EAAT 1 and EAAT 2 mRNAs were increased.

**Conclusion:**

We demonstrate that the CXCL12/CXCR4 signaling pathway contributes to the development and maintenance of neuropathic pain via central sensitization mechanisms. Importantly, intervening with CXCL12/CXCR4 presents an effective therapeutic approach to treat the neuropathic pain.

## 1 INTRODUCTION

Neuropathic pain is defined as pain arising from a lesion or disease affecting the somatosensory system either at peripheral or central level,^1^ and it is the major subtype of pathological pain. Neuropathic pain is manifested as spontaneous pain, hyperalgesia, allodynia, and secondary hyperalgesia. A systematic review demonstrated that neuropathic pain has a weighted average prevalence of 7% in adults, and is a hindrance to the activities of daily living and reduces job efficiency, which results in a poor quality of life.^2^ However, since the pathogenesis of neuropathic pain is still not fully understood, the diagnosis and treatment of neuropathic pain still faces enormous challenges.

Recent work has yielded a better understanding of the cellular and molecular mechanisms of neuropathic pain. Neural plasticity, which contains both peripheral sensitizations, mainly in the dorsal root ganglion (DRG), and central sensitizations in the spinal dorsal horn and supra‐spinal areas, is essential for the development and maintenance of neuropathic pain.^3^ Moreover, central sensitization mechanism has been considered to be the main target in the research of neuropathic pain. If peripheral sensitization after nerve lesions increases pain sensitivity, it is central sensitization, which could amplify pain and reduce threshold, causing neuropathic pain eventually.^4^ Spinal dorsal horn (SDH), as the primary portal for the integration of pain, has been implicated in this process. Central sensitization in spinal dorsal horn can be divided into two parts: plasticity of synaptic transmission and disinhibition.^5^, ^6^ Numerous mechanisms have been proposed in the plasticity of synaptic transmission, in which Glutamate/NMDA receptor‐mediated neuron sensitization,^6^ microglia and astrocyte activation–induced neuron‐glia crosstalk,^7^, ^8^ and the change of (pro‐inflammatory) cytokine microenvironment^3^ are considered as the major mechanisms. However, until very recently, the practical translation of the central sensitization theory to the design of new therapeutic approaches is lacking.

Stromal cell‐derived factor 1 (CXCL12/SDF‐1) belongs to the C‐X‐C subfamily of chemokine and exerts its effects via the CXCR4 receptor. In recent years, the role of the CXCL12/CXCR4 axis in pathological pain has been widely investigated. In a unilateral sciatic nerve injury model, an increase of CXCL12 and CXCR4 proteins was found in both L4‐L5 and C7‐C8 DRG,^9^ suggesting that they may participate in the peripheral sensitization of neuropathic pain. CXCL12/CXCR4 axis also plays an important role in central sensitization mechanisms of pathological pain. Intrathecal blockade of CXCL12/CXCR4 axis decreased the release of inflammatory cytokines, and attenuated ischemia‐reperfusion–induced inflammatory pain, which may be attributed to the inhibition of glial TLR4 activation in the spinal cord.^10^ CXCL12/CXCR4 signaling is involved in the development and maintenance of bone cancer pain via sensitizing neurons or activating astrocytes and microglia.^11^ CXCL12/CXCR4 axis also plays an important role in the neuropathic pain caused by peripheral nerve injury. In the pSNL model, CXCL12/CXCR4 signaling increased the production of pro‐inflammatory cytokines in the microglia, which triggered the development of neuropathic pain.^12^ In the SNI model, CXCL12 showed a long‐lasting upregulation in neurons and microglia, while CXCR4 was mainly increased in the neurons and astrocytes. These two factors ameliorate the established neuropathic pain and prevent its progression via activation of the ERK pathways.^13^ However, the key mechanisms accounting for the role of the CXCL12/CXCR4 axis in neuropathic pain remain unclear.

Considering the diversity and complexity of CXCL12/ CXCR4 axis in the regulation of neuropathic pain, we set out to investigate whether CXCL12 and CXCR4 are increased in SDH and participate in neuropathic pain using a rat SNL model. We demonstrate that CXCL12/CXCR4 signaling regulates the development and maintenance of neuropathic pain via central sensitization mechanisms. Interventions targeting the CXCL12/CXCR4 signaling axis are likely to be effective therapeutic approaches for neuropathic pain.

## 2 MATERIALS AND METHODS

### 2.1 Animals

Experiments were performed using adult male Sprague‐Dawley rats (body weight 180‐220 g). All rats were housed in separate cages and had free access to food and water with a natural light/dark cycle under conditions of 24 ± 1°C. All experimental protocols were in accordance with the guidelines of the International Association for the Study of Pain and were approved by the Animal Care and Use Committee of Soochow University.

Eighty‐four rats were used in the behavioral testing. The first two groups (n = 12) received SNL surgery for mechanical and thermal hypersensitivity testing, respectively. To study the effects of CXCL12 in the maintenance of neuropathic pain, rats were randomly divided into three groups and were subjected to SNL surgery. On postoperative day (POD) 7, the rats were treated with a single intrathecal injection of Anti‐CXCL12 neutralizing antibody (1 or 5 μg Anti‐CXCL12 + SNL group, n = 12) or of control IgG (5 μg IgG + SNL group, n = 6). To explore the effects of CXCL12 in the development of NP, the rats were randomly divided into two groups to receive either Anti‐CXCL12 neutralizing antibody (5 μg Anti‐CXCL12 + SNL group, n = 6) or control IgG (5 μg IgG + SNL group, n = 6) on POD 3 for three consecutive days. To demonstrate the effects of a single intrathecal injection of rat recombinant CXCL12 peptide on pain sensitivity, naive rats were treated with either 250 ng CXCL12 peptide (CXCL12 group, n = 6) or 20 μL 1% DMSO (1% DMSO group, n = 6). To explore the CXCR4 location cell‐dependence, naive rats received the corresponding cell inhibitors (cell inhibitors group, n = 6). To confirm the roles of CXCR4 in the development and maintenance of NP, rats were randomly divided into four groups to receive SNL surgery. On POD 3 or 7, they were treated with a consecutive intrathecal injection of 20 μg AMD3100 (AMD3100 + SNL POD 3 or POD 7 group, n = 12) or saline (saline + SNL POD 3 or POD 7 group, n = 12).

### 2.2 Drugs and antibodies

Rat recombinant CXCL12 peptide (Z02860) was purchased from Genscript (Piscataway, NJ, USA). CXCR4 antagonists AMD3100 (A5602) and Astrocyte metabolic inhibitor fluorocitrate (F9634) were purchased from Sigma (St. Louis, MO, USA). Anti‐CXCL12 neutralizing antibody (ab25117) and control IgG (ab133470) antibody were purchased from Abcam (Cambridge, MA, USA). AMD3100, Anti‐CXCL12 neutralizing antibody and control IgG were dissolved in sterilized saline. CXCL12 peptide and fluorocitrate were prepared in 1% dimethyl sulfoxide (DMSO).

Antibodies against GFAP (ab10062), CXCL12 (ab25117),^14^ CXCR4 (ab197203),^15^ NeuN (ab104224), and OX42 (ab1211) were purchased from Abcam. Antibodies against CXCR4 (11073‐2‐AP)^16^ were purchased from Proteintech (San Ying biotechnology, Wuhan, China). Antibody against Actin (sc‐8432) was purchased from Santa Cruz Biotechnology (Santa Cruz, CA, USA). Anti‐mouse IgG (H + L) (#4408) and anti‐Rabbit IgG (H + L) (#4413) were purchased from Cell Signaling Technology (CST) (Danvers, MA, USA).

### 2.3 SNL model

The SNL model is a representative animal model of peripheral neuropathic pain. Rats were anesthetized with sodium pentobarbital (30‐45 mg/kg, ip), and the left L6 transverse process was carefully removed to expose the L4 and L5 spinal nerves. The L5 spinal nerve was then isolated and tightly ligated with a 5‐0 silk thread.^17^ The incision was closed with a 3‐0 silk thread and disinfected with ethanol and iodophor. For the sham‐operated group, we removed the L6 transverse process, and then the L5 spinal nerve was only exposed and isolated without nerve ligation.

### 2.4 Behavioral analysis

Animals were habituated to the testing environment daily for at least two days before baseline testing. The temperature and humidity were kept stable for all experiments. Rats were placed in plastic chambers and allowed 30 minutes for acclimation before the examination. The mechanical or thermal stimulation was carried out three times to each hind paw at 5 minutes intervals.

### 2.5 Mechanical pain sensitivity

Mechanical hypersensitivity was assessed with Electronic Von Frey (e‐VF, No. 38450, Ugo Basile, Comerio, Italy), an electronic apparatus for applying a light touch to the rat's hind feet. Using a rigid metal tip, we applied a continuous force until the rats’ hind paw withdrawal was observed. The Ratemeter was used to monitor and ensure us that the desired force was applied at a consistent rate as 10 g/s. The e‐VF recorded the animal's response automatically and showed the maximum value when the rats suddenly released their paws. The maximum value was the paw withdrawal thresholds (PWT).

### 2.6 Thermal pain sensitivity

Thermal hypersensitivity was tested using the Plantar Test (Hargreaves Apparatus, No.37370, Ugo Basile, Comerio, Italy), an instrument which automatically detects the paw withdrawal latency (PWL). The protocol was similar to that described by Hargreaves et al.^18^ To prevent tissue damage, the basal paw withdrawal latency was adjusted to 9‐12 seconds and a cutoff of 25 seconds. The intensity of the heat stimulus was kept constant throughout the study.

### 2.7 Intrathecal injection

Intrathecal injection of drugs was performed based on a previously described protocol.^19^ Briefly, the rats were anesthetized with sodium pentobarbital. After shaving the hair, the lumbar region was disinfected with 75% (v/v) ethanol. Next, using a microliter syringe (Hamilton) with a 30‐gauge needle (BD), we delivered a total volume of 20 μL drug(s) to the subarachnoid space between the L4 and L5 levels. The needle was left in place for at least 10 seconds after injection. The injection area was disinfected with 75% (v/v) ethanol after the operation. The success of the administration was verified by a tail‐ or paw‐flick response immediately after inserting the needle. Rats with signs of motor dysfunction were excluded from the experiments.

### 2.8 Immunohistochemistry

After appropriate survival times, rats were deeply anesthetized with sodium pentobarbital and perfused with 0.9% saline followed by 4% paraformaldehyde in 0.1 mol/L phosphate buffer (PB) via the cardiovascular system. The L4/5 spinal segments were removed and post‐fixed overnight. After that, segments were dehydrated using graded sucrose (20% and 30%) in PB for at least two nights until they completely sunk to the bottom. Transverse spinal sections (30 μm) were cut on a cryostat and prepared for immunofluorescence staining. Sections were randomly selected and placed into different wells of 6‐well plates. After washing with PBS, the sections were first blocked with 0.2% BSA for 2 hours at 37℃, and then incubated overnight at 4℃ with the following primary antibodies: rabbit anti‐CXCL12 (1:500, Abcam), rabbit anti‐CXCR4 (1:500, Proteintech), Mouse anti‐GFAP (a marker for astrocytes, 1:1000, Abcam), Mouse anti‐NeuN (A neuronal marker, 1:1500, Abcam), and Mouse anti‐OX42 (a microglial marker, 1:500, Abcam). After rewarming, the sections were washed with PBS and incubated with the corresponding secondary antibodies conjugated with Alexa Fluor 488 or 555 (1:1000, CST) for 2 hours at 37℃. For double immunofluorescence staining, all the sections were incubated with a mixture of primary and secondary antibodies appropriately. The stained sections were examined and images were captured with a Nikon fluorescence microscope (DS‐Qi2, Nikon Co., Tokyo, Japan).

To obtain a quantitative analysis of GFAP and OX42 immunofluorescence in the spinal dorsal horn, the same fields covering spinal dorsal horn in each group were evaluated and photographed at the same exposure time to generate the raw data. The average green fluorescence intensity of each pixel was normalized to the background intensity in the same image. The immunofluorescent images were analyzed by Image J (NIH Image, Bethesda, MD, USA).

### 2.9 Real‐time PCR

After sacrificing each mouse with decapitation, the L4/5 section of its spinal dorsal horns was quickly removed and stored at −80℃ until RNA extraction. Total RNA was extracted by Trizol reagent (Takara, Shiga, Japan). One microgram of total RNA was converted into cDNA using PrimeScript RT reagent kit (Takara). The synthesized cDNA samples were diluted and stored at −80℃ until further testing. The cDNA was amplified using the specific primers shown in Table 1. All the primers were synthesized by Sangon Biotech (Sangon Biotech, Shanghai, China). The iTaq^TM^ universal SYBR Green Supermix (BIO‐RAD, USA) was used for all PCR reactions, which were run on an ABI Prism 7500 Fast sequence detection system (Applied Biosystems, Foster City, CA, USA). The PCR amplifications were performed at 95°C for 15 seconds, followed by 35 cycles at 95°C for 10 seconds, 60°C for 30 seconds, and 95°C for 15 seconds. The melting curves were performed to validate the utility and specificity of each PCR product. The ratio of mRNA expression relative to the control was evaluated using the Comparative CT Method.

**Table 1:** Primers for real‐time PCR

Gene	Primer	Sequence
TNF‐α	Forward	5′‐CCACGCTCTTCTGTCTACTG‐3′
	Reverse	5′‐GCTACGGGCTTGTCACTC‐3′
IL‐1β	Forward	5′‐TGTGATGTTCCCATTAGAC‐3′
	Reverse	5′‐AATACCACTTGTTGGCTTA‐3′
IL‐6	Forward	5′‐TGCCTTCTTGGGACTGAT‐3′
	Reverse	5′‐TTGCCATTGCACAACTCT‐3′
GFAP	Forward	5′‐GAGTGGTATCGGTCCAAGTT‐3′
	Reverse	5′‐CTCAAGGTCGCAGGTCAA‐3′
iba‐1	Forward	5′‐ATGAGCCAGAGCAAGGATT‐3′
	Reverse	5′‐GCATTCGCTTCAAGGACA‐3′
c‐FOS	Forward	5′‐TGTGACCTCCCTGGACTTG‐3′
	Reverse	5′‐CACTGGGCCTAGATGATGC‐3′
CGRP	Forward	5′‐GCGGGAAGAACAAGCATA‐3′
	Reverse	5′‐GGATCTCAACAGCGGTCA‐3′
Connexin 43	Forward	5′‐GTGACTGGAGTGCCTTGG‐3′
	Reverse	5′‐GTGGAGTAGGCTTGGACC‐3′
Connexin 30	Forward	5'‐AGACCTGGAGGACATCAAA‐3′
	Reverse	5'‐ACCCATTGTAGAGGAAGTAGA‐3′
EAAT 1	Forward	5'‐GTGCTTCGGCTTCGTGA‐3′
	Reverse	5'‐AGAGGATGCCCAGAGGTG‐3′
EAAT 2	Forward	5'‐GCCAAAGCACCGAAACCT‐3′
	Reverse	5'‐AAGCAGCCCGCCACATAC‐3′
GAPDH	Forward	5′‐GCAAGTTCAACGGCACAG‐3′
	Reverse	5′‐GCCAGTAGACTCCACGACAT‐3′

### 2.10 Western blot

Animals were sacrificed by decapitation and the L4/5 spinal dorsal horns were harvested and temporarily stored. Then, the samples were homogenized in ice‐cold RIPA lysis buffer. After measurement of concentration, the protein samples were separated on 10% SDS‐PAGE and electro‐transferred onto PVDF membranes. Protein samples were then incubated with antibodies for rabbit anti‐CXCL12 (1:1000; Abcam), rabbit anti‐CXCR4 (1:1000, Abcam), and mouse anti‐Actin (1:2000; Santa). The membranes were incubated with horseradish peroxidase–conjugated anti‐mouse or anti‐rabbit secondary antibodies (1:2000, Jackson), and exposed to film. The intensity of the selected bands was analyzed using Image J software.

### 2.11 Statistical analysis

The data were analyzed using GraphPad Prism, version 7.0 (GraphPad Software, Inc, San Diego, CA). Student's *t*‐test was used to analyze real‐time PCR data comparing differences between the AMD3100 group and saline group. Results from the immunohistochemistry and western blot, as well as alterations in the detected mRNA expression after SNL or CXCL12 peptide injection were tested using one‐way analysis of variance (ANOVA), followed by the Dunnett multiple comparison tests. Two‐way ANOVA with repeated measures followed by Bonferroni Multiple comparisons test were used to analyze data from the behavioral test. All data are presented as means ± SEM. A *P* value of <0.05 was considered to be statistically significant.

## 3 RESULTS

In all behavioral analysis experiments, six rats were used per group for each time point.

### 3.1 Mechanical and thermal hypersensitivity developed in the SNL model

**Figure 1:**
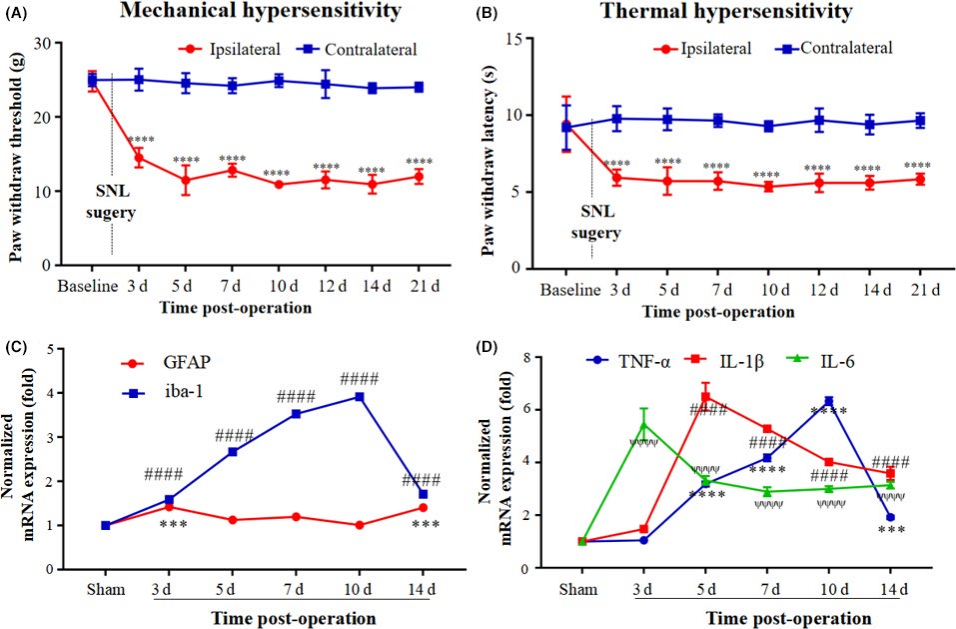
Neuropathic pain developement, glia activation, and upregulation of pro‐inflammatory cytokines in SNL‐injured rats. The PWT (A) and PWL (B) in the ipsilateral paw was decreased in the SNL model compared with contralateral paw. Results are means ± SEM. ****P < 0.0001 vs contralateral paw on POD 3 to 21. SNL also induced an obvious upregulation of GFAP, iba‐1 and pro‐inflammatory cytokines, as shown in C and D. Results are means ± SEM. ***P < 0.001, ****P < 0.0001, ####*P* < 0.0001, ^ѱѱѱѱ^ *P* < 0.0001 vs sham group

First, we constructed a stable SNL model in rat which was confirmed to be successful. Consistent with previous studies, SNL produced rapid and durable thermal and mechanical pain hypersensitivity in the left hind paws of rats. In addition, pain‐related behavioral analysis revealed a clear reduction of PWT (Figure 1A) and PWL (Figure 1B) from 3 to 21 days in the ipsilateral paw compared with the contralateral paw, which reflected a progressive development of neuropathic pain.

### 3.2 The relative mRNA expression of activated glia and pro‐inflammatory cytokines was increased after SNL

Glia activation and neuroinflammation are considered as important components of the central sensitization mechanism in neuropathic pain.^8^ We examined astrocytes and microglia activation by checking the mRNA level of GFAP and iba‐1 in the spinal cord after SNL treatment. We found that SNL induced a gradual upregulation of GFAP mRNA level in the spinal cord. The peak time was 2 weeks after surgery (Figure 1C). Compared with GFAP, the changes in iba‐1 expression were induced much earlier. Its level was increased at 3 days, and reached the peak at 10 days followed by a rapid downward trend at 14 days (Figure 1C). These results are in accord with those of a previous study in which microglia and astrocytes were separately found to be involved in the production and the maintenance of neuropathic pain.^8^ Besides glia activation, we also examined the expression profile of typical pro‐inflammatory cytokines. Real‐time PCR results showed that SNL induced a rapid increase in expression of TNF‐α, IL‐1β, and IL‐6 (Figure 1D). Interestingly, the mRNA level of TNF‐α increased at 3 days reaching the peak at 10 days, and followed by a rapid decline. Similarly, the peaked time of IL‐1β and IL‐6 was 3 and 5 days, respectively. As shown in Figure 1D, pro‐inflammatory cytokines mRNA levels remained at high levels at 2 weeks after SNL.

### 3.3 Temporal and spatial changes of CXCL12/CXCR4 in the spinal cord after the SNL‐induced neuropathic pain

**Figure 2:**
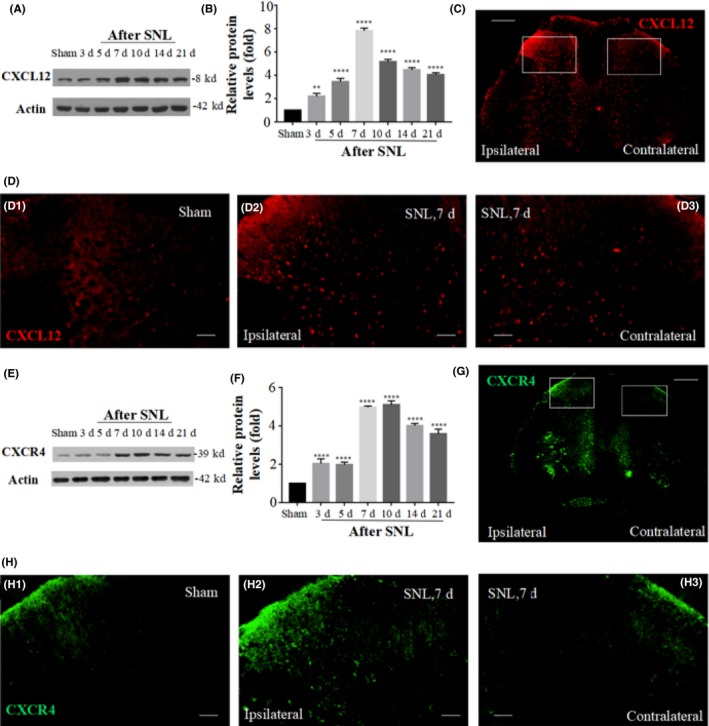
Expression and distribution of CXCL12 and CXCR4 protein in rat spinal cord after SNL. Western blot showed the time course of CXCL12 and CXCR4 expression in sham and SNL rats (A,E). Data analysis confirmed that SNL increased the CXCL12 and CXCR4 level in the SDH from days 3 to 21 after surgery (B,F). Results are means ± SEM. **P < 0.01, ****P < 0.0001 vs sham group. Immunofluorescence showed the distribution of CXCL12 (red) and CXCR4 (green) protein in the ipsilateral and contralateral spinal cord after sham or SNL surgery on POD 7 (C and G, scale bar, 500 μm). More details in spina dorsal horn were in D1, D2, and D3 or H1, H2, and H3 (scale bar, 100 μm)

Here, we examined whether CXCL12/CXCR4 chemokine signaling may be functionally upregulated in SNL‐induced neuropathic pain state. We first detected the expression and distribution of CXCL12 in the SDH after SNL. CXCL12 protein was found to be at a low level in the SDH of sham‐operated rats (Figure 2D1). SNL induced a rapid‐onset and long‐lasting expression of CXCL12 protein from day 3 to day 21 (Figure 2A). This increase was observed at day 3 which reached the peak at day 7 and remained at a high level until day 21 (Figure 2B). The immunohistochemistry results showed that CXCL12 was predominantly distributed in the ipsilateral laminae III‐V layers in SDH at day 7 after SNL (Figure 2D2,D3). But CXCL12 was not restricted to the SDH. A widespread distribution of CXCL12 was observed in the gray nucleus after SNL (Figure 2C).

The expression and distribution of CXCR4 were also explored. SNL induced an obvious increase in CXCR4 protein level from day 3 to day 21 after surgery (Figure 2E). Changes in its protein level were observed at day 3, and reached the peak at day 7 or day 10, and remained at a high level at day 21 (Figure 2F). Immunohistochemistry examination showed that CXCR4 was distributed mainly in the ipsilateral laminae I and III‐V layers of SDH, compared with the contralateral side (Figure 2G,H).

**Figure 3:**
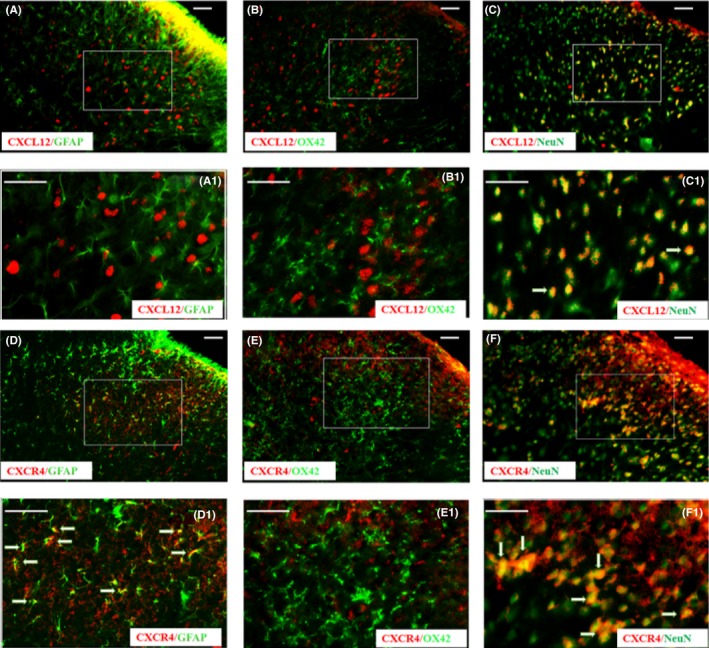
Cell localization of CXCL12 and CXCR4 in spinal dorsal horn of SNL rats. Double staining images A,B, and C showed that CXCL12 (red) is colocalized with NeuN (green, right), but not with GFAP (green, left) or OX42 (green, middle). while CXCR4 (red) is colocalized with GFAP and NeuN, but not with OX42, as shown in D, E, and F. More details are in A‐1 to F‐1.Spinal segments were collected 7 d after SNL (all images, scale bar, 100 μm)

To investigate the cellular localization of CXCL12/CXCR4, we performed a double immunofluorescent labeling of CXCL12/CXCR4 with three major spinal cell‐specific markers containing NeuN (for neurons), GFAP (for astrocytes), and OX42 (for microglia). CXCL12 was extensively colocalized with NeuN (Figure 3C,C‐1), but not with GFAP (Figure 3A,A‐1), or OX42 (Figure 3B,B‐1), suggesting that CXCL12 is induced by neurons, but not astrocytes or microglia in the rat after SNL. These results demonstrated that the CXCL12 was upregulated in the spinal cord after SNL and predominately expressed in excited neurons.

We further examined the cellular distribution of CXCR4 and found that CXCR4 was primarily localized in the neurons and astrocytes, but not in the microglia (Figure 3D,E,F, and D‐1,E‐1,F‐1). These results indicated that SNL activated neurons and astrocytes in the ipsilateral side of SDH, which further increased the expression of CXCR4.

### 3.4 CXCL12 modulated pain‐related hypersensitivity through central sensitization mechanism in naive rats

Following the observation that SNL could induce a significant upregulation of CXCL12. We further explored whether CXCL12 peptide could modulate pain‐related hypersensitivity in naive rats and the associated mechanisms. Normal rats were randomly divided into CXCL12 peptide group and 1% DMSO control group. The mechanical and thermal hypersensitivity were assessed after intrathecal injection.

**Figure 4:**
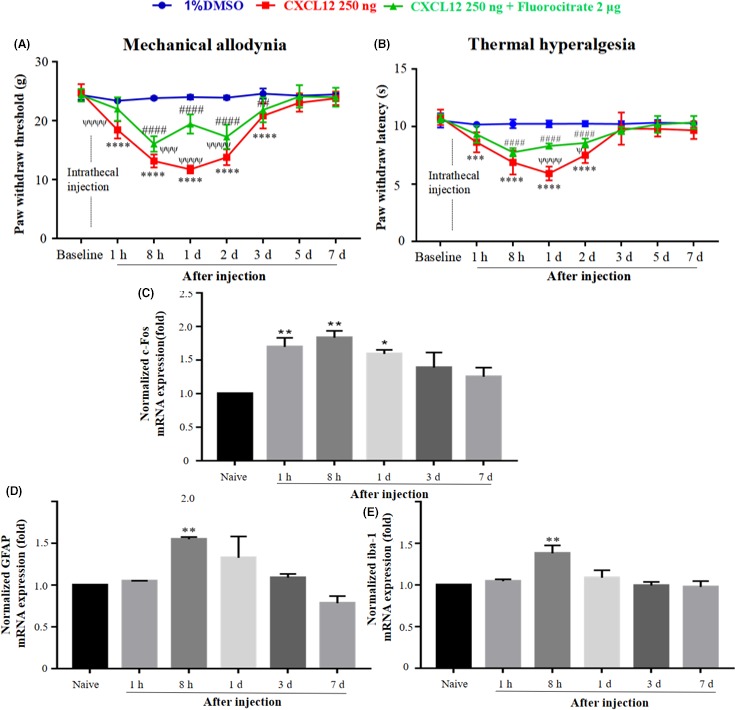
CXCL12 led to hypersensitivity by modulating the excitability of neurons and activation of astrocytes and microglia, which could be partially reversed by fluorocitrate in naive rats. As shown in A and B, a single intrathecal injection of rat CXCL12 peptide decreased both PWT and PWL of normal rats rapidly from 8 h to 3 d or 2 d, respectively. The cotreatment of CXCL12 with fluorocitrate prevented CXCL12‐induced hyperalgesia. The reversion sustained until CXCL12 gradually lost its function on nociception. Results are means ± SEM. ***P < 0.001, ****P < 0.0001 CXCL 12% vs 1% DMSO. ##*P* < 0.01, ####*P* < 0.0001 CXCL12 + fluorocitrate vs 1% DMSO. ^ѱ^ *P* < 0.05, ^ѱѱѱ^ *P* < 0.001, ^ѱѱѱѱ^ *P* < 0.0001 CXCL12 vs CXCL12 + fluorocitrate. The mRNA level of c‐Fos raised swiftly since 1 h, and the upregulation lasted until 1 d after injection (C). GFAP, iba‐1 levels began to go up until 8 h later and did not last for a long time (D and E). Results are means ± SEM. *P < 0.05, **P < 0.01, vs the naive group.

As shown in Figure 4A,B, the intrathecal delivery of 1% DMSO did not influence PWT and PWL in normal rats. However, a single intrathecal injection of rat CXCL12 peptide (250 ng) decreased PWT from 1 hour to 3 days and decreased bilateral hind paws PWL, which lasted for up to 2 days.

To confirm whether CXCL12 was involved in the central sensitization mechanism, we checked for the activation of neurons (c‐Fos),^20^, ^21^ astrocytes (GFAP), and microglia (iba‐1) after CXCL12 injection. The Real‐time PCR results showed that CXCL12 peptide injection triggered c‐Fos, GFAP, and iba‐1 expression. The results indicated that CXCL12 could directly modulate the sensitization of neurons and activation of astrocytes and microglia in naive rats (Figure 4C‐E).

### 3.5 The fluorocitrate partially reversed CXCL12‐mediated nociception in naive rats

Having confirmed that CXCR4 was predominately expressed in neuron and astrocyte, the cellular targets of neuronal CXCL12 in the neuropathic pain process were further investigated. Fluorocitrate, a metabolic inhibitor of astrocytes, was used for this purpose. CXCL12 rat peptide (250 ng) and fluorocitrate (2 mg) were co‐injected intrathecally to normal rats. Compared with the single CXCL12 peptide group, the co‐injection group showed a partial remission in pain‐related hypersensitivity. As shown in Figure 4A,B, co‐administration affected both mechanical and thermal hypersensitivity in the normal group, and the effect was sustained for at least 2 days. These results demonstrated that neuronal CXCL12 induced nociception through astrocytic CXCR4. Considering the finding that fluorocitrate could only partially reverse the pain perception caused by CXCL12 injection, we indirectly showed that the cellular targets of CXCL12 contain CXCR4 localized neurons as well.

### 3.6 Effect of intrathecal CXCL12 neutralizing antibody on the development and maintenance of neuropathic pain in the SNL model

**Figure 5:**
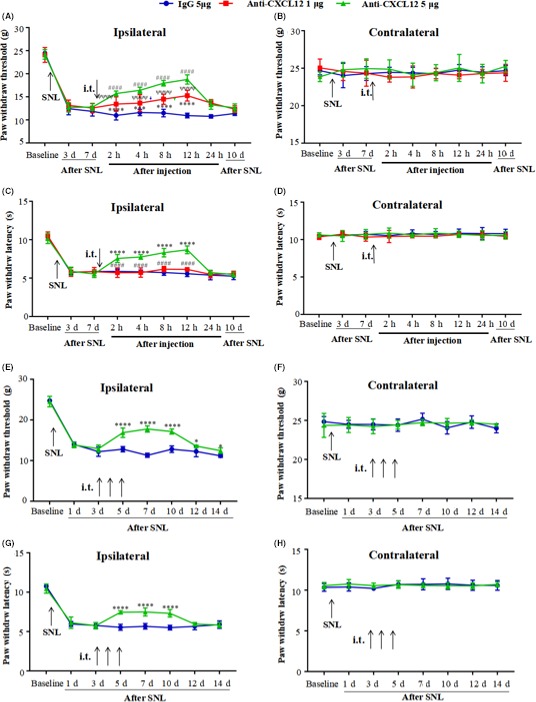
Intrathecal administration of CXCL12‐neutralizing antibody attenuated and prevented neuropathic pain after SNL in rats. A single intrathecal administration of CXCL12‐neutralizing antibody (1 μg/20 μL, 5 μg/20 μL, it) in the later phase (POD 7) suppressed the ongoing mechanical hypersensitivity in ipsilateral side for 12 h in SNL rats (A). However, 1 μg CXCL12‐neutralizing antibody could not affect thermal hypersensitivity, compared to the 5 μg CXCL12‐neutralizing antibody group (C). Repeated intrathecal administration of CXCL12‐neutralizing antibody (5 μg/20 μL, i.t.) in the early phase (POD 3, 4 and 5) partially prevented the development of both mechanical and thermal hypersensitivity in SNL rats (E,G), which could maintain until POD 14. Besides, the treatment did not affect the contralateral hind paw in either mechanical or thermal pain sensitivity (B,D,F,H). Results are means ± SEM. For Figure 5A, ***P < 0.001, ****P < 0.0001 1 μg CXCL12‐neutralizing antibody vs 5 μg IgG. ####*P* < 0.0001 5 μg CXCL12‐neutralizing antibody vs IgG. ^ѱѱѱѱ^ *P* < 0.0001 1 μg vs 5 μg CXCL12‐neutralizing antibody. For Figure 5C, ****P < 0.0001 5 μg CXCL12‐neutralizing antibody vs IgG. ####*P* < 0.0001 1 μg vs 5 μg CXCL12‐neutralizing antibody. For Figure 5E,G, *P < 0.05, ****P < 0.0001 5 μg CXCL12‐neutralizing antibody vs IgG

We first investigated the effect of SNL‐induced upregulation of CXCL12 in the established neuropathic pain model. The rats received a single intrathecal injection of 1 μg or 5 μg CXCL12‐neutralizing antibody on day 7 after SNL, at which time the neuropathic pain had fully developed. The behavioral tests revealed that the CXCL12‐neutralizing antibody partially reversed the reduction of PWT and PWL in SNL rats in a dose‐ and time‐dependent manner. Compared with the control IgG group, 5 μg CXCL12‐neutralizing antibody significantly increased the PWT and PWL for 12 hours, but the 1 μg dose could only affect the PWT (Figure 5A,C). Interestingly, the treatment did not affect either the mechanical or thermal pain sensitivity in the contralateral hind paw after SNL (Figure 5B,D).

To evaluate the role of CXCL12 in the development of neuropathic pain, 5 μg CXCL12‐neutralizing antibody was injected it for 3 consecutive days at day 3 after SNL. Compared with the IgG group, the 5 μg CXCL12‐neutralizing antibody partially prevented the SNL‐induced neuropathic pain. The difference of PWT between 5 μg CXCL12‐neutralizing antibody and control IgG persisted until day 14; 9 days after administration (Figure 5E). Likewise, the difference of PWL persisted for 5 days after administration (Figure 5G). However, the consecutive it treatment did not affect the contralateral hind paw in terms of mechanical and thermal pain sensitivity development after SNL (Figure 5F,H).

### 3.7 Blocking CXCL12 suppressed SNL‐induced activation of microglia cells and astrocytes

**Figure 6:**
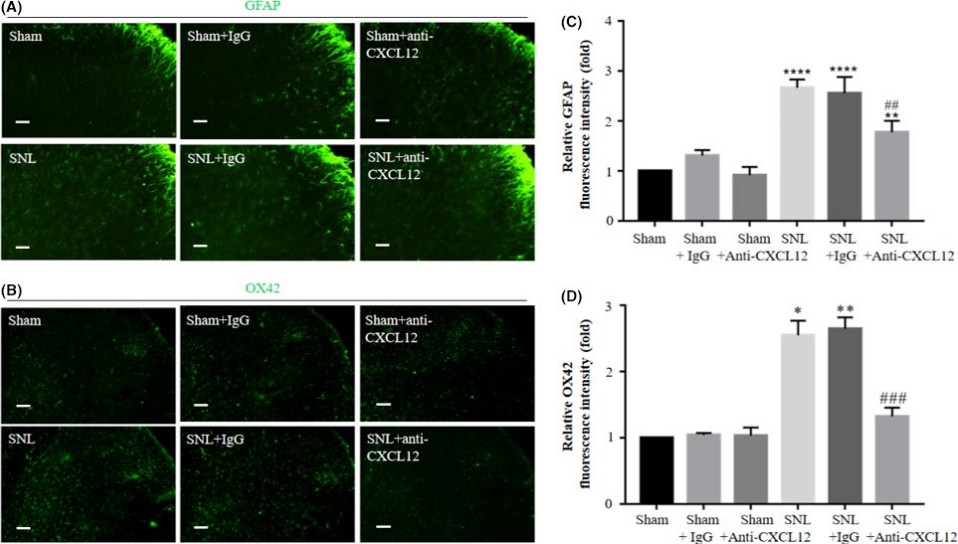
CXCL12‐neutralizing antibody suppressed SNL‐induced upregulation of GFAP and OX42 expression in spinal cord dorsal horn. Immunofluorescence micrographs (A and B, scale bar, 100 μm) showed activation of astrocytes (GFAP) and microglia (OX42) caused by SNL, compared with the sham group. These changes were suppressed by successive intrathecal injection of CXCL12‐neutralizing antibody but not control IgG. In the meantime, the treatment did not influence the sham operation group. Data summary further confirmed these results, as shown in Figure 6C,D. Tissues were collected 1d after the last injection. Results are mean ± SEM. *P < 0.05, **P < 0.01, ****P < 0.0001 SNL vs sham. ##*P* < 0.01, ###*P* < 0.001 SNL vs SNL + anti‐CXCL12

Spinal nerve ligation caused glia cells activation in the spinal cord as shown in Figure 1C. To reveal the cellular consequences of CXCL12 upregulation, we further investigated whether CXCL12 may be involved in the activation of astrocytes (GFAP) and microglia (OX42) caused by SNL. The immunofluorescence images showed that SNL caused significant elevation of GFAP and OX42 protein expression compared to the sham group, which was in line with the mRNA level analysis. SNL‐mediated induction of astrocytes activation (Figure 6A,C) and microglia (Figure 6B,D) was remarkably suppressed by the CXCL12‐neutralizing antibody (5 μg/20 μL it, once a day from day 3 to day 5 after SNL) relative to the control IgG. However, in the sham group, neither CXCL12‐neutralizing antibody nor the control IgG changed the expression of GFAP and OX42. These results indicated that spinal CXCL12 may directly modulate the activation of astrocytes and microglia in the SNL model.

### 3.8 Effects of intrathecal AMD3100 injection on the development and maintenance of neuropathic pain in the SNL model

**Figure 7:**
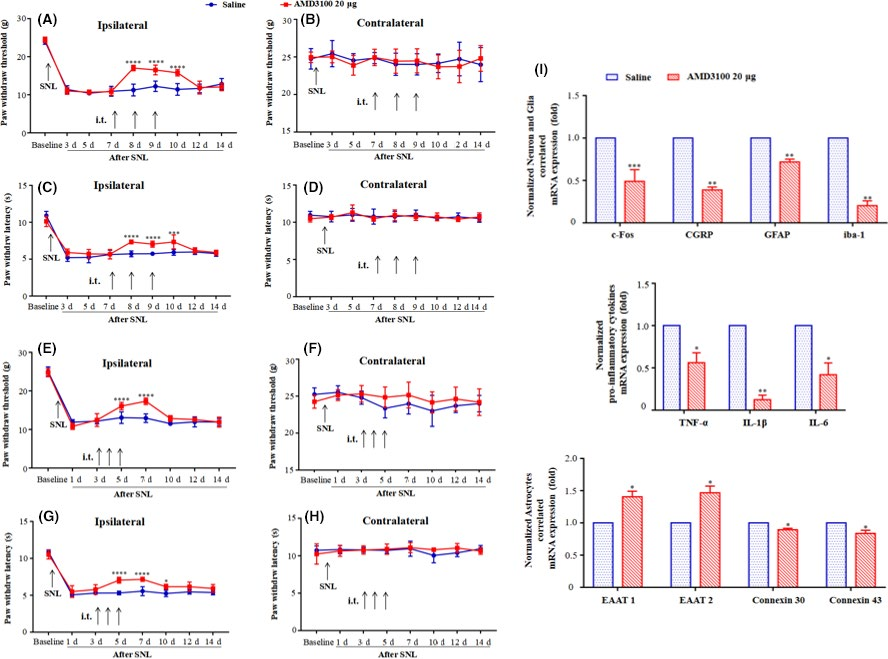
The effects of intrathecal AMD3100 injection on the maintenance and development of neuropathic pain in SNL‐injured rats via central sensitization mechanisms and gila‐neuron interaction. The intrathecal AMD3100, but not saline, at POD 7 or POD 3 after SNL surgery, increased ipsilateral PWT and PWL of SNL rats after the injection (A,C and E,G), but did not affect contralateral side (B,D and F,H). Real‐time PCR (I) revealed that intrathecal AMD3100 decreased the mRNA level of the c‐Fos and GFAP and iba‐1, and suppressed pro‐inflammatory cytokines (TNF‐α, IL‐1β, IL‐6) mRNA expression. Besides, neuron‐correlated CGRP and astrocyte‐correlated Connexin 30, Connexin 43 were decreased, while EAAT 1 and EAAT 2 mRNAs were increased after intrathecal AMD3100 injection. Results are mean ± SEM. *P < 0.05, **P < 0.01, ***P < 0.001, ****P < 0.0001 vs the saline group

To explore the role of central CXCR4 in the maintenance of neuropathic pain, SNL‐injured rats received either continuous intrathecal AMD3100 or saline starting from day 7 after SNL for three days. Mechanical or thermal hypersensitivity was assessed until day 14 after SNL. Intrathecal 20 μg AMD3100 increased the ipsilateral PWT and PWL from day 7 to 10 after the injection (Figure 7A,C). Saline did not have any effect on the ipsilateral pain sensitivity, and no significant difference was observed in the contralateral PWT or PWL between the AMD3100 and saline groups (Figure 7B,D). These results suggest that intrathecal AMD3100 may transiently reverse an established neuropathic pain caused by SNL injury.

We further investigated whether CXCR4 was involved in the development of neuropathic pain. AMD3100 or saline was given intrathecally at day 3 after SNL. The behavioral analyses demonstrated that AMD3100 had a significant effect on the ipsilateral PWT and PWL when compared with the saline group. Interestingly, intrathecal AMD3100 showed a prolonged inhibitory effect on thermal hypersensitivity than mechanical hypersensitivity (Figure 7E,G). No difference in contralateral PWT or PWL was found (Figure 7F,H). These results suggest that intrathecal AMD3100 may delay the development of neuropathic pain in the SNL model.

### 3.9 Blocking CXCR4 prevented and alleviated the SNL‐induced neuropathic pain by suppressing central sensitization and gila‐neuron interaction

As the main component of central sensitization in the spinal dorsal horn, plasticity synaptic transmission is affected by neuronal sensitization, microglia, and astrocyte activation. Both neuronal and glial cells, together with blood‐borne macrophages, play critical roles in the induction and maintenance of neuropathic pain by releasing potent neuromodulators, such as pro‐inflammatory cytokines, which enhance neuronal excitability.^22^ Therefore, we investigated whether intrathecal AMD3100 could affect SNL‐induced neuropathic pain by regulating the excitability of the spinal neuron and glia cells and the progress of neuroinflammation in the spinal cord. We found that the mRNA levels of c‐Fos, a representative marker of neuronal sensitization,^21^ were significantly decreased in SNL‐injured rats treated with intrathecal AMD3100 compared to the saline group at 1 day following repeated injection starting from day 3 to 5 after SNL (Figure 7I). Besides, the same phenomenon was observed in the mRNA expression of GFAP, iba‐1, and pro‐inflammatory cytokines (TNF‐α, IL‐1β, and IL‐6) after AMD3100 treatment (Figure 7I). The Real‐time PCR data indicated that CXCR4 contributed to SNL‐induced neuropathic pain via suppressing central sensitization.

Since AMD3100 showed a prolonged inhibitory effect on thermal hypersensitivity than mechanical hypersensitivity, we further detected the mRNA level of CGRP (calcitonin gene‐related peptide), a marker of neurons essential for heat responses,^23^ and found that the mRNA level of AMD3100 group was lower. Considering CXCR4 was partially colocalized with astrocytes, we investigated whether intrathecal AMD3100 could modulate characteristic astrocyte‐related factors. We found that the mRNA levels of EAAT 1 and EAAT 2 were significantly increased, while the gap junction channels Connexin 30 and Connexin 43 were decreased in AMD3100 group compared to the saline group.

## 4 DISCUSSION

The role of the CXCL12/CXCR4 axis in pathological pain has been extensively studied, but its mechanism is multifarious and complex. In this study, we first demonstrated that CXCL12 was remarkably upregulated in active neurons after SNL. Blocking CXCL12 attenuated pain hypersensitivity, probably by suppressing the activation of microglia and astrocytes. Moreover, CXCL12 peptide showed the ability to modulate pain‐related hypersensitivity via the central sensitization mechanism in naive rat. On the other hand, CXCR4 was increased in neurons and astrocytes, and CXCR4 antagonist attenuated neuropathic pain by regulating central sensitization and gila‐neuron interaction. Finally, decreasing CXCR4 by inhibiting astrocyte metabolism partially reversed the CXCL12‐induced hypersensitivity. Taken together, these findings suggest that the CXCL12/CXCR4 axis plays an important role in the development and maintenance of neuropathic pain, which points to its potential therapeutic utility to treat neuropathic pain.

### 4.1 CXCL12 is upregulated in the activated neurons of the spinal cord and is involved in the regulation of neuropathic pain

Recent studies have shown that the expression profile of CXCL12 in the spinal cord is quite diverse among different pain models. Ischemia/reperfusion or tumor cell implantation may trigger a prolonged upregulation of CXCL12.^10^ In this study, the CXCL12 protein levels were progressively increased after injury and reached the peak at day 7. These results indicate that CXCL12 may be involved in neuropathic pain development.

Central sensitization is dependent on the enhanced functional status of neurons, which is in turn caused by increased membrane excitability, synaptic efficacy, leading to enhanced nociceptive processing and hyperalgesia.^5^, ^6^ There has been an overwhelming research focused on the involvement of neuronal‐secreted chemokines or their receptors in the central sensitization regulation and pathological pain. In the SNL model, CCR2 and CXCR2 in the spinal neurons may change neuronal properties by activating the ERK signaling, which phosphorylates the NMDA receptor and enhances synaptic transmission.^24^ Recently, as a novel regulator of neuropathic pain, CXCL12, was found to be upregulated in the spinal neurons after anti‐tubulin chemotherapeutics in rat.^25^ The planter incision model induces CXCL12 upregulation in both neurons and astrocytes.^26^ Similarly, in our study, we found that CXCL12 was produced by spinal neurons after SNL in male rats, and that intrathecal injection of CXCL12 neutralizing antibody alleviated neuropathic pain at both the development and maintenance phase. Moreover, intrathecal injection of rat CXCL12 peptide influenced the mechanical pain and thermal pain sensitivity in normal rats. Finally, we confirmed that CXCL12 affected neuropathic pain, partly, by influencing the sensitization of neurons. Taken together, these studies suggest that CXCL12 may be considered as one of the neurotransmitters that regulate neuropathic pain through direct neuronal central sensitization mechanism.

Recent studies have shown that neighboring astrocytes and microglia are powerful modulators of pain. Neuron‐glia interactions or glia‐mediated central sensitization mechanisms also play critical roles in neuropathic pain.^27^ Through the BDNF/trkB‐KCC2 axis, activated microglia drive neurons to a wide dynamic range status, providing the cellular substrate for neuropathic pain.^28^, ^29^ Astrocytes are by far the most abundant glial cells within the central nervous system. In peripheral neuropathic pain models, astrocytes shifted to a “reactive” phenotype, released a high number of factors such as NO, PGs, excitatory amino acids, and ATP, suggesting that astrocytes may be involved in pain maintenance.^30^ Accumulating evidence has indicated that chemokines play an important role in neuropathic pain through neuronal‐glial interaction. CX3CL1 is secreted by primary afferents and spinal neurons and binds to its receptor CX3CR1 to induce microglial activation.^31^ CXCL13 secreted by spinal neurons was found to regulate neuropathic pain via promoting astrocytes activation in SNL mice through its receptor CXCR5.^32^ This further supported the preposition that chemokines and their receptors participate in the central sensitization mechanisms by indirect mechanisms. In our study, we confirmed that CXCL12 influenced neuropathic pain by triggering the activation of astrocytes and microglia in naive rats. Furthermore, the CXCL12‐neutralizing antibody eliminated the upregulation of GFAP and iba‐1 in SNL rats. These data indicate that CXCL12 may regulate sense perception through glia‐mediated central sensitization mechanisms.

### 4.2 Effects of CXCR4 expressed in activated neurons and astrocytes on neuropathic pain

Evidence suggests that CXCR4 is associated with different pathological pain. Blocking CXCR4 at the peripheral level or its knockdown was shown to prevent bee venom‐induced inflammatory pain state and primary mechanical and thermal hypersensitivity.^33^, ^34^ Here, we found that blocking CXCR4 with its antagonist, AMD3100, delayed the development of neuropathic pain and reversed the established mechanical and thermal hypersensitivity induced by SNL in rats. These findings are consistent with the results found in partial sciatic nerve ligation (pSNL) mice.^35^ Collectively, these results confirm that CXCR4 may play a significant role in the peripheral nerve injury‐induced neuropathic pain.

To explore the specific central sensitization mechanism involved in neuropathic pain, cellular localization of CXCR4 was determined. Similar to its ligand, CXCR4 shows diverse distribution in the spinal dorsal horn in different pathological pain conditions. CXCR4 exclusively colocalized with neuronal cells in the Plantar incision‐induced postsurgical pain.^26^ But, CXCR4 was predominantly localized to the astrocytes and microglia in a rat model of ischemia/reperfusion‐induced inflammatory pain.^10^ In our study, SNL induced notable increase of CXCR4, which was predominantly in the neurons and astrocytes of the spinal dorsal horn. This is in agreement with the results reported in spared nerve injury rats.^13^ We further showed that blocking CXCR4 with AMD3100 influenced the excitatory state of neurons as well as the activation of astrocytes, indicating that CXCR4 participated in the central sensitization mechanism and hence modulated neuropathic pain. Particularly, we found AMD3100 shows a prolonged inhibitory effect on thermal hypersensitivity. CGRP (Calcitonin gene‐related peptide) is a classic molecular marker of peptidergic primary somatosensory neurons. The variable responses to noxious heat are due to strain‐dependence of CGRP expression and sensitivity.^36^ Genetic ablation of CGRPα‐expressing sensory neurons could reduce sensitivity to noxious heat and impaire thermoregulation.^37^ Similarly, we found that the mRNA level of CGRP was obvious decreased after AMD3100 injection. Nevertheless, whether CXCR4 directly activates some intracellular pathways to alter the functions of these cells should be investigated in further studies.

Except neuronal plasticity and glia activation, it has been recognized that, as pivotal mediators of immune and inflammatory reactions, pro‐inflammatory cytokines released by resident glia or infiltrated macrophages and T cells may also contribute to the central sensitization processes and regulate pathological pain.^8^ TNF‐α/TNFR1 signaling has been found to regulate synaptic plasticity in lamina I neurons, resulting in heat hyperalgesia and thermal hypersensitivity after complete Freund's adjuvant intraplantar injection.^38^ IL‐1β increased the excitability of superficial dorsal horn neurons by enhancing AMPA and NMDA responses in the substantia gelatinosa (SG) neurons.^39^ IL‐6 has also been implicated in neuropathic pain caused by multiple aetiologies. In red nucleus (RN), IL‐6 participates in SNI‐induced neuropathic pain through activating JAK/STAT3 and ERK signaling pathways.^40^ In addition, several recent studies suggest that the effects of pro‐inflammatory cytokines on neuronal excitability may be mediated via an indirect mechanism. Both behavioral and electrophysiological effects of IL‐1β are absent following the disruption of glial cell activity.^41^ Glia was found as the source of the majority of intrathecal TNF‐α and IL‐1β, IL‐6 that accompanies mechanical hypersensitivity in the chronic constriction injury rat.^42^ Here, we found that intrathecal injection of CXCR4 antagonist suppressed the SNL‐induced mRNA upregulation of TNF‐a, IL‐1β, and IL‐6, indicating that CXCR4 may prevent the SNL‐induced neuropathic pain through the pro‐inflammatory cytokines‐dependent central sensitization mechanism.

Interestingly, AMD3100 was found to modulate the excitatory state of microglia. It is widely accepted that microglial cells trigger the production of pro‐inflammatory cytokines, such as TNF‐α, IL‐1β, and IL‐6 via the p38/MAPK (mitogen‐activated protein kinase) signaling pathway, which in turn regulate synaptic transmission in the superficial spinal dorsal horn.^28^ Hence, considering that microglial cells are a major source of pro‐inflammatory cytokines in the spinal cord dorsal horn, we postulated that CXCR4 may influence pro‐inflammatory cytokines, at least partially, due to its effect on microglia activation.

### 4.3 CXCL12/CXCR4 signaling modulates astroglial‐neuronal interaction in neuropathic pain

CXCL12 and CXCR4 are expressed in the neurons and astrocytes, and glial‐neuronal interaction has been implicated in the central sensitization under pathological conditions.^24^, ^43^ We therefore explored the specific cellular mechanisms related to the CXCL12/CXCR4‐mediated nociception.

First, blocking CXCL12 ameliorated the GFAP upregulation after SNL. It has been shown that reactive astrocytes sustain the pain condition by releasing factors such as cytokine and chemokine receptors that facilitate nociception.^8^ Astrocytic connexin‐43 has been implicated in gap junction and hemichannel communication of cytosolic contents, and played an essential role in maintaining late‐phase neuropathic pain by inducing chemokine release from astrocytes in mice.^44^ Additionally, glutamate transporters, EAAT 2 and EAAT 1, expressed in astrocytes may also regulate extracellular glutamate concentrations and elicit nociceptive hypersensitivity.^45^, ^46^ We assumed that increased CXCL12 may impact neuropathic pain via activating and affecting CXCR4‐localized astrocytes. Herein, we found that blocking CXCR4 by antagonist AMD3100 could raise EAAT 2 and EAAT 1 mRNA expression, and decrease the Connexin 43 and Connexin 30‐Hemichannel Activity in spinal cord astrocytes. Furthermore, the CXCL12 peptide activated the CXCR4 localized in the neurons and astrocytes leading to pain‐related hypersensitivity in naive rats, which is in line with previous studies.^12^ Thirdly, decreasing CXCR4 by inhibiting astrocyte metabolism may partially prevent CXCL12‐induced neuropathic pain. In other words, the CXCR4 in astrocytes accounted for some of the CXCL12‐induced hypersensitivity, indirectly suggesting that neuronal CXCR4 may be considered as an important component of the CXCL12‐mediated nociception. Taken together, our behavioral and molecular experiments have revealed important patterns of the CXCL12/CXCR4 axis‐mediated astroglial‐neuronal crosstalk in the naive rats and SNL model.

In conclusion, this study suggests that CXCL12/CXCR4‐mediated astroglial‐neuronal interaction contributes to not only the occurrence but also the maintenance of neuropathic pain through central sensitization mechanisms in SNL or naive rats. CXCL12/CXCR4 signaling may serve as a novel target that can be exploited for the treatment of peripheral nerve injury‐induced neuropathic pain.

## CONFLICT OF INTEREST

The authors declare no conflict of interest.
